# Impact of Online Advertisement on Customer Satisfaction With the Mediating Effect of Brand Knowledge

**DOI:** 10.3389/fpsyg.2022.919656

**Published:** 2022-07-01

**Authors:** Anas A. Salameh, Mahrukh Ijaz, Abdullah Bin Omar, Hafiz Muhammad Zia ul Haq

**Affiliations:** ^1^Department of Management Information Systems, College of Business Administration Prince Sattam Bin Abdulaziz University, Al-Kharj, Saudi Arabia; ^2^Institute of Banking and Finance, Bahauddin Zakariya University, Multan, Pakistan; ^3^Department of Business Administration, National College of Business Administration and Economics (NCBA&E), Lahore, Pakistan

**Keywords:** online advertisement, brand knowledge, customer satisfaction, Pakistan, social media marketing

## Abstract

The purpose of this article is to explain how online advertising affects customer satisfaction through the mediation of brand knowledge. The sample size of this survey is based on 100 participants in the Multan region. This study collects data by conducting various unstructured interviews. In this study, we used a qualitative data acquisition technique. The results show that online advertising does not have a significant impact on customer satisfaction. However, when brand knowledge is included as a parameter, the correlation between online advertising and customer satisfaction increases. Online advertising is a new advertising tool used by most organizations. This manuscript helps practitioners choose better tools for online promotion and uses a variety of recognition techniques to improve their brand knowledge. It has been known through this study, that building customer confidence in product quality is a very effective approach in front of business owners, as brand reputation enhances customer satisfaction. This study is unique in that previous studies considered elements of brand knowledge as parameters and ignored to find a direct relationship between online advertising and customer satisfaction. This study highlights key points that will help emerging researchers critically analyze such aspects in future studies.

## Introduction

There is a growing need for knowledge and information regarding the online purchasing behavior of consumers due to radical change in e-commerce, which is also known as electronic commerce (Bhakar et al., 2019). In the current era, it becomes a dynamic concept of how organizations manage their customer relationships and what kind of marketing strategy should be adopted for the customer (Ahmad et al., 2022). Nowadays, the internet is not only used for information purposes but also as a platform for buying and selling goods and services between the buyer and seller (Danish et al., 2016). To survive in this highly diverse market, it becomes essential for organizations to adopt different marketing tools through the internet to attract their customers. The preference level of the customer is continuously changing due to the higher technological influence on their living pattern (Islam and Rahman, 2017). Being a seller, it becomes crucial to consider the importance of technology in their operating, marketing, and sales department (Gul et al., 2022). Thus, in the current digital era, online shopping has become a fascinating variable for the management and entrepreneurs to gain market share and customer satisfaction in the competitor’s market and also to secure the future of a company (Nwokah and Ngirika, 2017). Some researchers also depicted that the perfect knowledge regarding customer preference data plays an important role in creating a direct and long-term relationship with them. Thus, most multinationals and SMEs worked on making online advertisements to attract many customers in a short period (Mehmood and Sabeeh, 2018).

According to the survey results, most of the customers are not only searching the products on the internet for purchasing but most of them are interested to gain some important information about the specific products (Rabeea Fatima et al., 2019). Unfortunately, most products advertised on social media, differ from their actual look. There are many scamming advertisements on social media that negatively affects the reputation of the company (Rabeea Fatima et al., 2019). Also, it affects badly the confidence level of the customers and, as a result, a high dissatisfaction level exists among the consumers regarding online buying and fake advertisements (Ismail and Alawamleh, 2017). People usually purchase goods or services online and enter their personal information on web sites, which increases the numbers of cyber crimes (Ismail and Alawamleh, 2017).

This manuscript is informative to investigate the impact of an online advertisement about the products and services on the satisfaction level of the customer, where a sustainable brand knowledge (i.e., perception level of a customer about the brand) plays a mediating role between these two variables (Kim et al., 2017). In Pakistan, consumers shift their behavior toward e-commerce because of the technological influence on their daily transactions (Danish et al., 2016). In this manuscript, we specifically highlight the satisfaction level of Pakistani customers with online advertisements and evaluate how sustainable knowledge about the brands plays an important role in keeping the satisfaction level of the customer regarding the company’s operation. No previous study exists to critically evaluate the relationship between online advertisement and customer satisfaction, which enhanced the validity of this research work (Srivastava and Chopra, 2016). Ultimately, the study will help customers and consumers in assisting them in online purchase decisions and the corporate sector in formulating their marketing strategies. The current study also helps the practitioners to choose the better tools for an online advertisement and also increase the brand knowledge by using different awareness techniques. This manuscript also fulfills the gap in the previous related literature regarding online advertisement’s impact on customer satisfaction and the role of sustainable brand knowledge on their relationship.

## Literature Review

Previously, no study was majorly conducted by scholars to comprehend the impact of an online advertisement on the satisfaction level of the customer, by considering sustainable brand knowledge as a mediator. To justify this research, some similar research made by the previous scholars is discussed below, which will enhance the authenticity and reliability of this research.

### Online Advertisement and Customer Satisfaction

Many researchers have worked to elaborate on the direct relationship between online advertisement and customer satisfaction levels. According to Hanif and Asgher (2018), an online advertisement depicts that now, the majority of the advertisements are occurring through the internet by using different platforms, like Email, YouTube, Facebook, Instagram, Daraz.com, and other advertisement supporting websites (Hanif and Asgher, 2018). According to them, an online advertisement is one of the most significant marketing tools in today’s digital world, even though most organizations do not think of capturing the market share without advertisement. They stated that in the current era, the style of marketing research is becoming modernized because of the impact of upgraded communication technology on the advertisement factor which adds value to the buyer’s choice (Hanif et al., 2018a). The effect of knowledge management and entrepreneurial orientation on organization performance.

The histrionic increases the scope and diversity of online advertisement than conformist one. In stated businesses are extensively using such virtual advertisements to promote versatile products and services. Mediating Role of Supply Chain Integration and Intrapreneurship between Information Technology Infrastructure and Firm Performance in the Iranian Pistachio Industry. They concluded this one is hard for an advertiser to maintain the effectiveness of online advertisements to get a positive reaction from consumers (Ahmad and Gul, 2021; Bukhari et al., 2021b).

According to Nazli et al. (2018), the growth of media and communication networks has altered the business landscape of advertising, so now an online advertisement becomes an essential approach to increase the profit margin of a company (Nazli et al., 2018). Advertising properties, such as the design, quality, duration, or location of an advertisement, can affect the effectiveness of such ads. The scholars concluded that such an attractive source of marketing helps a company to maintain its position in the market.

According to the researchers (2017), e-commerce promised a “perfect” arrival in the market by introducing product and price comparison websites, the so-called shopping robots (Phillips et al., 2017). They stated that technology is about to achieve what economists could only undertake in the past: “near-perfect information.” Nunan et al. (2018) stated that customer satisfaction is an accumulated attitude based on his or her experiences. They stated that there must be a feeling of a customer that can be gauged directly. Customer satisfaction is directly related to a firm’s profitability. Customers may be satisfied by different factors, e.g., product design, product advertisement, etc. (Nunan et al., 2018). These researchers point out the three obstacles to online shopping namely failure to buy, security, and service fear hindrances. At the end of their critical analysis, they concluded that more and more people have to shop online or indeed provide information to web providers for access to information (Ali et al., 2021; Gul et al., 2021b).

In Ghazali et al. (2016) stated that a deprived account of the online customer experience is based on a 24% loss in annual online revenue, more than $50 billion losses in the United States, and a £14 billion lost in the United Kingdom each year (Ghazali et al., 2016). According to them, a basic strategic aim for many firms is to upgrade the customer’s experience level and the perception level regarding the company’s products and services will ultimately affect the firm’s profitability. For this purpose, customer satisfaction is a crucial factor in any firm’s existence.

### Brand Knowledge

In 2019, research was conducted based on critically evaluating the importance of brand knowledge and its importance in enhancing the brand image in the customer market. According to Anusha (2019), brand knowledge is a newly introduced term, which refers to the experiences, thoughts, and feelings of a customer regarding the brand and its operating activities. According to the researcher, one of the most important objectives of product and brand management is to build a powerful brand and its loyalty factor within a customer market. This research concluded that strong brand results in greater income streams, both in short term and long term (Gul et al., 2021a).

Scholars conducted another research to evaluate the two major types of brand knowledge: brand awareness and image. According to Von Wallpach and Kreuzer (2019), two dimensions of brand knowledge have been previously examined in marketing research. The first one is brand awareness and the second one is the brand image. According to them, brand awareness is defined as the strong point of product bulge in memory, i.e., how easy it is for the customer to recall that brand (Bukhari et al., 2021a). They have a point of view that brand recall is the most common way to measure brand awareness. They stated that comprising many brand knowledge and behavioral variables, using one product category like “candy bars,” plays an important role in strengthening a customer’s and company’s relationship. In 2019, the researchers started support for a two-factor solution: one factor represented the unassisted recall (Cheung et al., 2019). They stated that the brand image creates a strong, favorable, and unique brand reputation in the mind of the customers and other stakeholders.

In 2020, after conducting critical qualitative research on this factor, the researchers concluded that the perceived quality, positive attitude, and overall profitability ratio in a company are generated due to enhancing the standard of marketing and sales channels (Cheung et al., 2020). The influence of perceived social media marketing elements on consumer–brand engagement and brand knowledge. They stated that many of the brand factors acknowledged as diverse facets of brand parity by other scholars (for instance, apparent quality, character, and organizational associations) may be fit into the overall classification of brand image and its abrupt effects. Another researcher examined the moderating effect of brand knowledge definitely to the two variables (brand awareness and brand image) (Suki, 2016).

In 2018, a study on online advertisement and customer satisfaction depicted that brands used different advertisement channels to attract their customer as well as to satisfy their needs and desires (Hanif et al., 2018b). Nowadays, it is impossible to capture market share and satisfy their customer without advertisement. For this purpose, brands used different media for advertisement, and in today’s world, online advertisements through social media and other websites, etc., are very popular to deliver products to their customers (Hanif et al., 2017). Finally, they concluded that brand knowledge is also a very important factor that mediates the relationship between online advertisement and customer satisfaction because the individual’s perceptions and feelings about their brand matter a lot in customer satisfaction.

### Brand Knowledge Strengthens the Relationship Between Online Advertisement and Customer Satisfaction

In 2017, studies were conducted by researchers to critically evaluate the importance of the co-creational factor of the brand in strengthening the relationship between the online brand’s communities and customer satisfaction (Hajli et al., 2017). According to them, a positive perception regarding the company’s products and services in the targeted customer market plays an important role in creating the long-term sustainability of a company in the customer market. In their business research market, they concluded that a positive word of mouth and an understanding of the company’s operation play an important role to gain a competitive advantage in the highly competitive market.

In another related research article (Gul et al., 2020a,b), scholars majorly worked to critically evaluate the importance of the productive brand knowledge in the development of customer perception level regarding the company’s products and services (Cheung et al., 2020). The influence of perceived social media marketing elements on consumer–brand engagement and brand knowledge. They concluded that such knowledge, which developed through some experiences, boosts the confidence level of the customers to use the services and products of a brand. According to the researchers, it is an easy way to strengthen a good relationship between the company and its targeted customers (Adarsha et al., 2021; Irfan, 2021).

Based on the synthesized review of literature, previous studies do not have a consensus on finding regarding the relationship among said variables and the significance of an online advertisement on customer satisfaction. Although some studies address the same topic, to the best of the authors’ knowledge, none of the studies considers the same issue by incorporating the mediating effect of brand knowledge, especially in a developing market context; hence, this area is still under-researched. Therefore, this study intends to fill this gap by conducting unstructured interviews to examine the impact of an online advertisement on customer satisfaction.

## Materials and Methods

To understand the impact of online advertisements on customer satisfaction and sustainable brand knowledge, which mediates the relationship between the independent variable (IV) and dependent variable (DV), different unstructured interviews and other observations based on secondary data were collected. The questionnaire used by Teo, T with others in their research article is used for this analysis (Teo et al., 2018). Responses were collected through formal and informal but unstructured interviews, observations, and directly asked questions to respondents.

The population of this research was based on households, students, officers, hoteliers, and other related people. The sample was based on 100 individuals, who were chosen from several areas of the Multan region. For this purpose, questions were asked to exactly understand the impact of an online advertisement on their satisfaction and also about their knowledge regarding brands’ advertisements and online shopping sites where they purchased some goods. All this data collection is based on formal or informal interviews and other online secondary information. Each interview was held for only 20–25 min to better understand the participant’s perception level regarding online advertisements. As discussed earlier, the interview format is highly unstructured.

This qualitative technique is more effective for this research analysis because the base of this research was focused on critically evaluating the psychological and behavioral approach of the targeted audience of any companies in the current era (Gul and Khilji, 2021). So, for that purpose, firstly we distributed questionnaires to the respondent, but the response rate is not good and the collected data are full of errors as people show less interest in filling the questionnaires. Also, some questions are not properly interpreted by respondents. In that research survey, 100 responses were collected and from which 60 responses were understandable, so the response rate was 60%. Questions about demographic variables such as age and gender are considered controlling variables.

This study used a cross-sectional data collection method because all responses were collected at once. Convenient sampling is also used for data collection. Data were analyzed through personal interpretations and analytical models. It is an effective research method for critically inspecting and evaluating the cognitive characteristic of a common person regarding the importance of such an online advertisement in the Pakistan market.

### Conceptual Framework of the Study


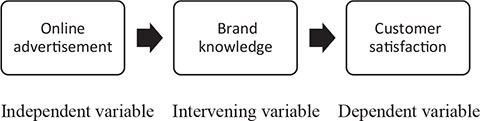


### Hypothesis

By reviewing the literature and critically evaluating the relationship between these variables, we have developed the following major hypothesis of this research work:

*H1:* Online advertisements have a significant impact on customer satisfaction.

*H2:* Brand knowledge has a significant and positive impact on customer satisfaction.

*H3:* Brand knowledge strengthens the relationship between online advertisement and customer satisfaction.

## Results and Discussion

This survey analysis uses several elements in this survey interview and analysis process. Some of the important questions for respondents are listed in Appendix. The questions in these surveys asked demographic information such as age and gender-related data queried to respondents and considered control variables. Main theme, principal nodes, and child nodes are given in Table 1. In addition, respondents were asked a variety of questions related to elements of online advertising, customer satisfaction, and sustainable brand knowledge to understand the customer’s perspective on online advertising. For example, whether online advertising primarily follows the customer’s decisions and what kind of advertising the customer prefers. According to survey data, most corporate customers are impressed with the online data presented by the organization. This is a constructive approach to critically understanding the level of customer similarity and preference by establishing a feedback-based relationship with the customer. As mentioned earlier, this analysis uses a qualitative approach to critically assess the customer’s level of perception of the hypothesis of choice.

**TABLE 1:** Main theme, principal nodes, and child nodes.

Main theme	Principal nodes	Child nodes
Online advertisement impact on customer satisfaction, refereeing the brand knowledge	Online advertisement and customer satisfaction	Surfing the product
	Brand knowledge	Surety of good quality product
		Product description
		Product reviews
		Inconstant experience
		Activity for spare time

These are the questions asked from the respondents through unstructured interviews and observed the behavior of consumers online.

### Respondents’ Results

Consider the variables of online surfing. Here, we asked the respondents three questions that explain their general attitude toward online surfing. Respondents have different views on this factor, so most respondents say they spend a lot of time browsing ads for their products and services. This behavior is the same whether the brand is known to the customer or not. The results show that customers often spend a lot of time browsing products and services for more detailed information. As one respondent said:

*“Buying products online is an important issue, so we spend a lot of time finding more information, whether we know a particular brand or not.”*

Another respondent said that:

*“When I see the online advertisement of the known brand, I am certain that the product is not of poor quality.”*

It simply means that it is available on the online site and has a good product quality guarantee known to the customer. The second general brand knowledge is used to determine the impact of brand knowledge on customer satisfaction. Questions about this factor determine a customer’s choice, so customers are convinced that the best way to buy is to browse ads for their well-known brands. After searching for the product or service that best suits their needs, they purchased the product. The survey questions focused primarily on the level of customer awareness of advertising. According to the results, most people searched for different brands and preferred only those brands whose features and prices directly matched the desired value.

The final question of this factor is primarily based on whether a well-known brand delivered the product to the customer as shown in the online advertisement. The consequences of this factor go in the same direction. This shows that general brand knowledge about a product or service is of little concern when deciding to buy online. In Pakistan, people do not like to buy online because of the potential risk of loss. Because the response rate is neutral, well-known brands do not encourage customers to buy online products, but they guarantee that the products are of high quality.

One respondent answered that “There is too much lose…” By:

*“If you blindly buy a product without reading the entire description or review, you are already using that particular brand of a product and you can get the high-quality product as shown in the picture. I do not think so*… *It’s impossible.”*

It represents the customer’s attitude toward online advertising and online purchases. That is, customers buy online products from well-known brands rather than unfamiliar brands. According to the result data, brand knowledge plays an important role in building long-term relationships with customers. According to the respondents, they prefer to buy products and services of this brand that have a good reputation in the consumer market. This is to increase the customer’s confidence in purchasing the product. This is a useful resource for critically assessing the direct impact of a company’s positive reputation on the customer market.

As with the third element, they were asked about people’s interest in displaying online ads. Whether to consider online advertising as a source of information and not purchase products or services? The overall response to this factor was informative, as people do not like to shop online, but it also focuses on seeing online advertising for informational purposes. Most respondents say they frequently check emails sent by different brands to evaluate the brand and its product offerings. As one respondent said:

*“It is interesting to see a colorful advertisement not only when I bought that product; but yes, for the sake of information, maybe in future I want to buy these types of products.”*

Another said:

*“It is a pleasurable activity to see product advertisements and check your junk mails full of advertisements when you have nothing to do.”*

This shows that people are not very interested in buying online but are interested in online advertising for informational purposes. The above two answers and other related answers show that most customers prefer to get a lot of relevant information about products offered through online sources. According to them, most buyers only bought products that meet their needs and desires at a reasonable price, and the Internet is an easy and reliable source for them to get relevant information about their products and services.

Finally, customer satisfaction factors were critically discussed in interviews that ask respondents seven questions. This element asks about the price of online purchases, and the quality of products purchased on online sites, and asks questions related to respondents. This was an important source of critical information about the importance of online advertising for customer satisfaction in the market. Most of them have a neutral response to this factor, and some of them have a positive response to such a satisfaction factor. As one respondent said:

*“There are times when the online shopping experience is good and times when it is bad, but I think that items ordered from cheap online sites must have a more unpleasant experience than high-priced items.”*

According to the answers, the response rate of participants to a company’s products and services may deviate from the average position. By interpreting the data, we can know that prices can also be a factor in influencing customer satisfaction when purchasing online products. The reason is that price factors add some value to a company’s products, especially for price-sensitive customers. In the market, most customers prefer products that are very reasonably priced compared to other products. Considering demographic factors such as age and gender as control variables makes it very easy to access an individual’s behavioral approach. Most Multan-Pakistan online customers prefer to buy products and services that have a good reputation in the market. The market for Multan is considerably lower than in other cities in Pakistan such as Lahore, Islamabad, Karachi, and Faisalabad. But now, with the penetration of technology in the region, many educated people prefer to get relevant information about a particular product or service of the brand. Brand knowledge and reputation in the customer market play an important role in maintaining the position in the customer market.

The customers are completely unsatisfied or dissatisfied with the online purchase. Everyone shared their level of awareness, understanding, and experience. It shows the diversity of Multan’s customer market, as most people simply visit the brand’s online site to get relevant information about products, services, prices, features, and other relevant information. The reason is that people have different economic standards and most of them are online brands (Badar and Irfan, 2018; Ali and Zafar, 2021). Due to the low interest of people in such online promotions, the target markets in the region are only attracted through electronic and print media. After critically evaluating all the responses from the target audience, the majority of Multani’s customers are fascinated by their fruitful and engaging knowledge of each brand and its services. As in the region, only these brands make a profit by not only leveraging online marketing sources but also working on electronic, printing, and other digital media sources to generate positive awareness and word of mouth in their target markets (Zhang et al., 2013). Overall, online advertising does not have a big impact on customer satisfaction, but brand knowledge does.

The responses weaken the first hypothesis of this research that an online advertisement does not have a significant impact on customer satisfaction. But, their collected data justified the second hypothesis that brand knowledge has a significant and positive impact on the customer satisfaction level. The reason is that the word of mouth regarding the operating activities of any brand creates some knowledge and confidence level among the targeted customers regarding the company’s product and services (Irfan and Khar, 2021; Irfan and Shahid, 2021). Also, the above research data justified the third hypothesis of this research that brand knowledge plays a major role in strengthening the relationship between online advertisement and the customer satisfaction level (Zhu et al., 2021). The reason is that such brand knowledge-based mediating factors help the customer create some positive and negative perceptions regarding the brand’s products and services (Zhang et al., 2012). In most cases, such brand knowledge is usually developed when the customer gets some experience regarding the brand. According to the respondent, only those customers who will reuse the services of online shopping have some good experience and have some surety and reliability of their brand services.

## Conclusion

After critically assessing the impact of online advertising on client customer satisfaction by conducting qualitative research, brand knowledge plays an important role in improving a company’s performance level in the competitor’s market. Analytical results show that this is an era of information technology, and online advertising and online purchases play a key role in maintaining a company’s outstanding reputation in the customer and competitor markets over the long term. In this article, we concluded that online advertising has a direct impact on customer satisfaction because brand knowledge acts as an intermediary.

Online advertising does not significantly affect customer satisfaction with online purchases. However, customers who have positive knowledge of the brand for a particular product have a high level of customer satisfaction in the market. People tend to view online advertising as an important source of information, not for sale or purchase purposes. From this, we can conclude that brand knowledge, positive or negative, has a significant impact on customer satisfaction with the company. This factor also further enhances the interaction between online advertising and customer satisfaction. Otherwise, online advertising will not have a significant impact on customer satisfaction and brand awareness. This is an important study for critically assessing customer behavior by considering the importance of brand knowledge as a key parameter.

### Limitations and Future Directions

There are still some caveats with this survey as it is a productive survey aimed at clarifying the factors behind customer intrusion. The first is based on demographic variables such as income level, education level, religion, marital status, mortality rate, average family size, birth rate, the average age at marriage, and occupation. These control factors were not considered due to time limitations. Second, this survey was based on Multani’s customer perceptions of online advertising. There was no comparison or contrast presented to customer awareness in other developed regions. The future direction of the researchers is to critically assess the current market conditions in the region and assess the behavioral approaches of different customers in different regions of Pakistan and in other countries.

## Data Availability Statement

The original contributions presented in this study are included in the article/Supplementary Material, further inquiries can be directed to the corresponding author.

## Author Contributions

AS designed the analysis. MI collected the data. AO performed the analysis. HZ contributed analysis tools. All authors contributed to the article and approved the submitted version.

## Conflict of Interest

The authors declare that the research was conducted in the absence of any commercial or financial relationships that could be construed as a potential conflict of interest.

## Publisher’s Note

All claims expressed in this article are solely those of the authors and do not necessarily represent those of their affiliated organizations, or those of the publisher, the editors and the reviewers. Any product that may be evaluated in this article, or claim that may be made by its manufacturer, is not guaranteed or endorsed by the publisher.
