# Supplementary material for: Impact of Online Advertisement on Customer Satisfaction With the Mediating Effect of Brand Knowledge
Source: Front Psychol. 2022 Jul 1;13:919656. doi: 10.3389/fpsyg.2022.919656 (PMC9286041; doi:10.3389/fpsyg.2022.919656)
Supplement: Supplementary file 1 [file Table_1.docx]

***Appendix***

| **Factors** | **Label** |
| --- | --- |
| **1. Online Surfing** | I spend a lot of time surfing before I decide on an online purchase. |
|  | I made fewer visits to the site of the brand before the purchase of a product online. |
|  | I spent a lot of time surfing the websites for information about online products. |
| **2. Overall Brand Knowledge** | By searching for more information about the known brand, I am certain of making the best buy. |
|  | It pays to surf around before purchasing online. |
|  | I learned which products are suitable for me for my known brand by surfing around. |
|  | There is too much to lose by being ignorant about the products of known brands when I have purchased online. |
|  | Surfing around various sites helped me to find a lower price when I purchase online, like whether the brand is known or unknown. |
|  | I got it exactly from the known brand I purchased by seeing an online advertisement. |
| **3. Interest in e-Advertisement** | I like to browse online advertisements even when I don't plan to buy products. |
|  | I surf various online advertisements just to find out more about new products and styles. |
|  | I rarely read an advertisement that just seems to contain a lot of information. |
|  | I enjoy sampling different online advertisements for common products for the sake of comparison. |
|  | I read even my junk mail just to know about product descriptions in online advertisements. |
|  | I usually delete mail advertisements without reading them. |
|  | I don't care to find out what brand names of online products my friends have purchased. |
|  | I often read online advertisements just out of curiosity. |
| **4. Customer Satisfaction** | My attitude about purchasing online is favourable. |
|  | Considering everything, I think purchasing online is an excellent deal. |
|  | Purchasing online is desirable. |
|  | Considering the price product purchased online are of excellent quality for the price. |
|  | Purchased products match the perceived expectation that was created after seeing online advertisements. |
|  | I am confident buy online is an excellent decision. |
|  | The price of products online is very acceptable. |
